# Cauda Equina Syndrome in a Patient with Intradural Schwannoma at the Same Level as an Acute L2 Compression Fracture

**DOI:** 10.7759/cureus.5492

**Published:** 2019-08-26

**Authors:** Danny Mallol, Rossy Taveras, Jason Hartman, Michelle Granville, Robert E Jacobson

**Affiliations:** 1 Neurosurgery, Clinica Union Medica, Santiago, DOM; 2 Neurosurgery, Centro Cardiorenal del Cibao (CENCARCI), Santiago, DOM; 3 Pain Medicine, Spine and Orthopedic Center, Santa Barbara, USA; 4 Neurosurgery, University of Miami Hospital, Miami, USA

**Keywords:** intradural schwannoma, incidental spinal tumors, vertebral compression fractures, cauda equina syndrome, multilevel spinal fixation

## Abstract

Intradural tumors are found often as either incidental findings or during evaluation during magnetic resonance imaging (MRI) for lumbar and/or radicular pain. This patient presented with an acute L2 compression fracture, however, the initial MRI identified a large spinal mass separate from the fracture but at the same level. The patient had acute upper lumbar pain after a fall but the neurologic examination also revealed findings of early cauda equina syndrome with muscle weakness, asymmetric leg numbness, and urinary incontinence. Further history revealed the patient had been using a cane for several months and having difficulty walking with some upper lumbar pain but had not seen a physician. The differential was an extruded disc associated with the fractured endplate versus a tumor. Because of the neurologic symptoms, emergency open decompression combined with multilevel screw fixation was performed. At the time of the laminectomy, the dura bulged posteriorly, no ventral disc was found, and a 3-cm intradural schwannoma was successfully excised with rapid neurologic recovery. The article will review the relationship of cauda equina syndrome with osteoporotic fractures and the rarity of actual true disc extrusion with compression fractures, as well as the more common relationship of finding cauda equina syndrome with intradural tumors when there is severe canal stenosis as seen in this unusual case.

## Introduction

The discovery of spinal tumors and other intra and extraspinal abnormalities during a radiologic examination being performed for other reasons is well-recognized and common with the use of magnetic resonance imaging (MRI) in the routine work-up of patients for lumbar and/or radicular pain [1]. In a large series of MRI studies, tumors were incidentally found in only 0.05% [2]. Often, after an injury, and especially with fractures, computerized tomography (CT) or MRI scans are performed and other spinal pathologies, such as Tarlov cysts, vertebral hemangiomas, lumbar stenosis, and, rarely, unsuspected tumors can be found [1,3]. Tumors can present with back pain, with or without neurologic symptoms, including radicular complaints and, especially when the tumor is large, cauda equina syndrome (CES) [4]. With spinal tumors, there is often a significant time delay between the onset of symptoms and diagnosis because of the insidiousness of the symptoms that can mimic the back pain and leg complaints seen with general degenerative back complaints, with the average time to diagnosis just for lumbar tumors averaging eight months [5]. Patients with osteoporotic spinal fractures, which are found in the elderly population, have a significant incidence of lumbar stenosis with possible symptoms of neurogenic claudication, which can be confused with CES, although neurogenic claudication symptoms are more intermittent and positional and rarely associated with urinary incontinence [6]. Low force osteoporotic traumatic fractures rarely present initially with either radicular symptoms or CES but can develop these symptoms as a result of progressive kyphotic deformity and gradual stenosis of the canal in the fractured area [7]. However, forceful traumatic fractures with bone deformation and displacement of the apophyseal ring and endplate with disc extrusion can lead to spinal cord injury and conus or cauda equina injury [8]. This is an unusual case, with a clinical history that retrospectively revealed the patient was in the early stages of the development of CES from an unsuspected intraspinal tumor. This included gait imbalance and unilateral leg weakness that contributed to her fall, leading to an acute compression fracture and subsequent severe spinal canal compression followed by worsening of her neurologic symptoms and signs.

## Case presentation

**Figure 1:**
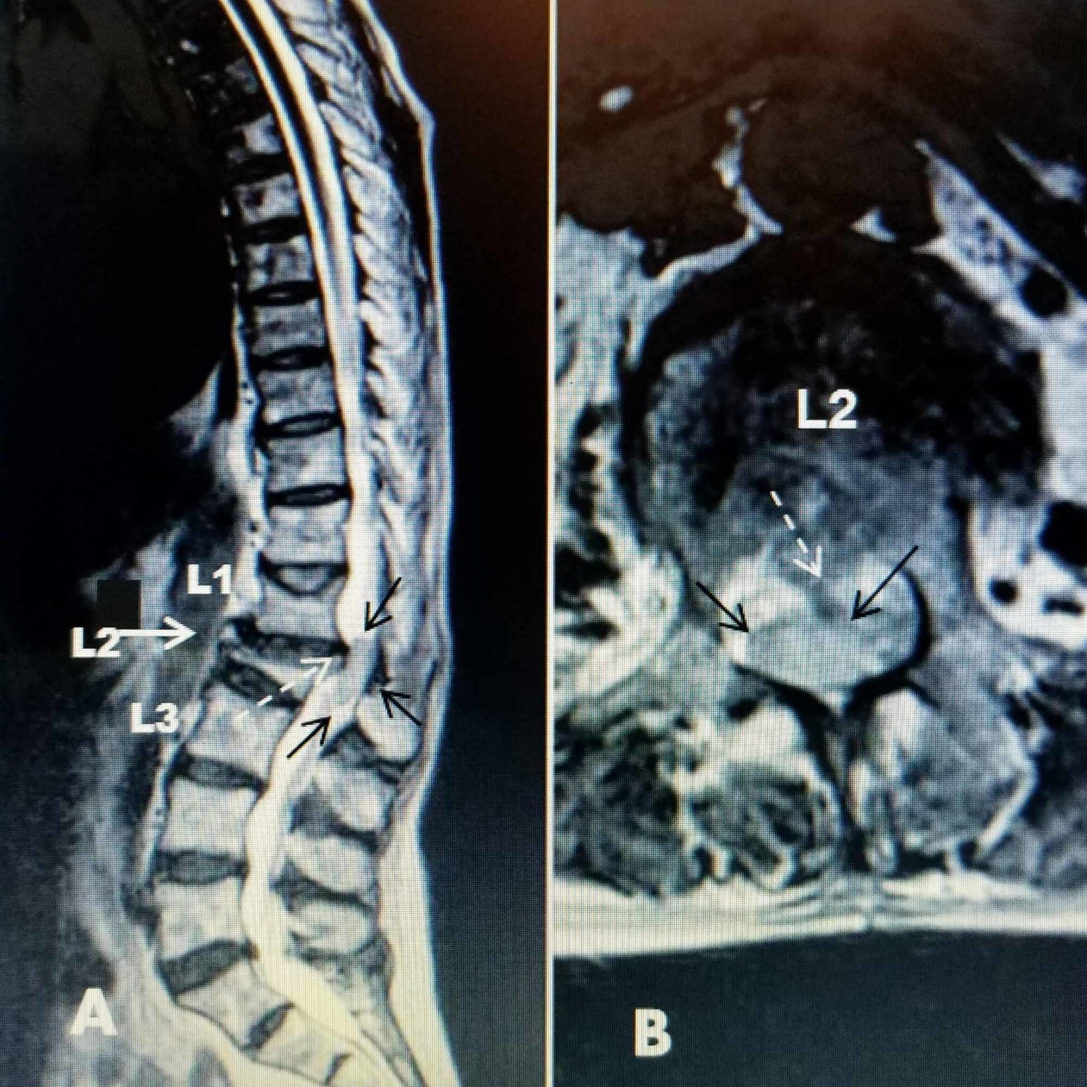
Initial MRI scan showing the L2 acute compression fracture and an intradural mass A: Sagittal spinal magnetic resonance imaging (MRI) showing an L2 70% wedge compression fracture (solid white arrow) with superior endplate edema and posterior displacement of 33% of the superior part of L2 (dashed white arrow) into the spinal canal. There is also minor superior endplate compression of L1. The isodense mass within the intradural space directly behind the L1-L2 disc space is clearly seen (solid black arrows) and, combined with the displaced endplate, causes almost 100% canal obstruction. B: Axial image at the level of the pedicles of L2 showing the homogeneous posterior intradural mass across the entire dorsal half of the spinal canal (solid black arrows). No conus or nerve roots are identified. The midline dorsally displaced posterior edge of L2 is seen just ventral to the intradural mass but comprising less than 50% of the axial compression (dashed white arrow).

A 62-year-old female presented to the emergency department with acute back pain and leg weakness, worse on the right, after falling at home. History revealed she had gait imbalance for over one month, had been using a cane, and complained of numbness in both feet with intermittent urinary incontinence prior to the fall. After the fall, her leg weakness immediately worsened. She had an emergency MRI performed showing severe compression at the level of the fracture at L2 and posterior displacement of the superior endplate of L2, with 20 degrees of kyphotic angulation, but there was also a large homogeneous isodense spinal mass at the same level (Figure 1).

**Figure 2:**
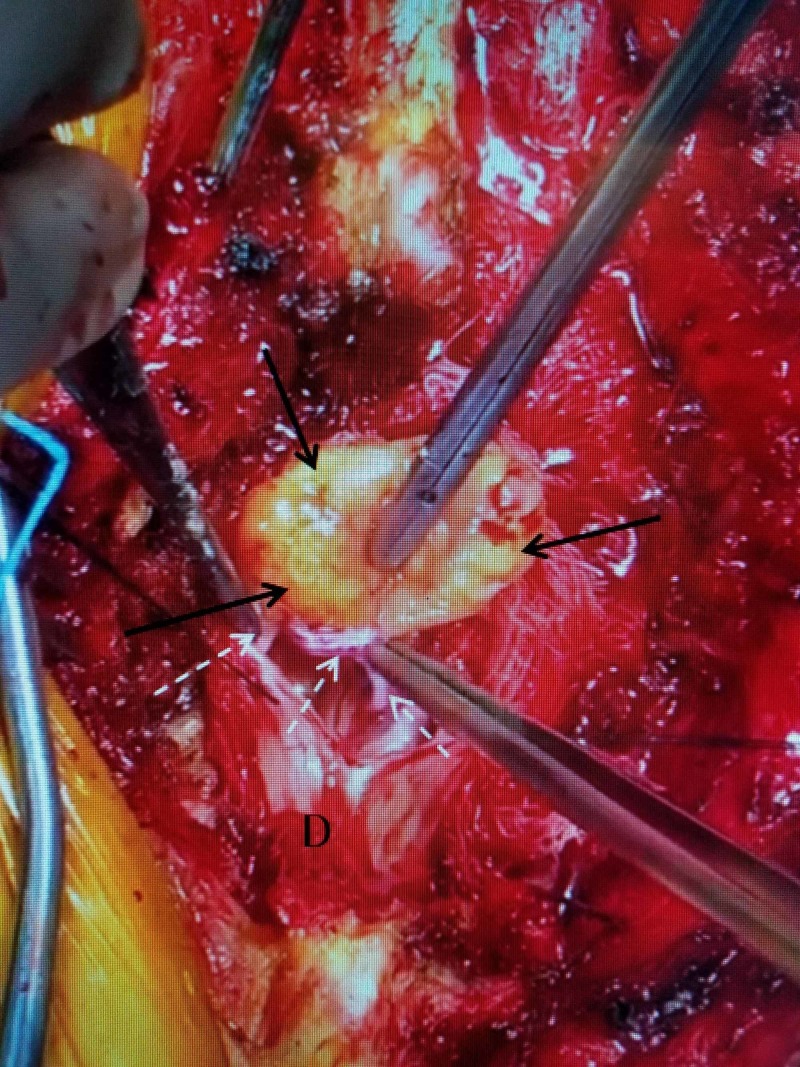
Intraoperative photo showing the removal of the intradural tumor The tumor was 30 x 18 millimeters (mm), yellowish, and firm (solid black arrows), with an intraspinal root attached inferiorly (dashed white arrows). The roots of the cauda equina were inflamed and matted below the tumor.

Because of the cauda equina syndrome and almost complete blocks seen on MRI myelogram, the patient underwent posterior decompression with planned multilevel pedicle fixation. At surgery, during a wide laminectomy, initially suspecting an extruded migrated disc and ruptured endplate, it was noted that there was posterior displacement of the dura. Retraction did not reveal any ventral dural mass except for fluoroscopic-confirmed superior endplate of L2, as seen on the MRI. The dura was incised and a yellowish, firm, 30 mm x 18 mm intradural tumor was identified, attached to several inflamed roots of the cauda equina. The tumor was removed in total with a water-tight sutured dural closure (Figure 2).

**Figure 3:**
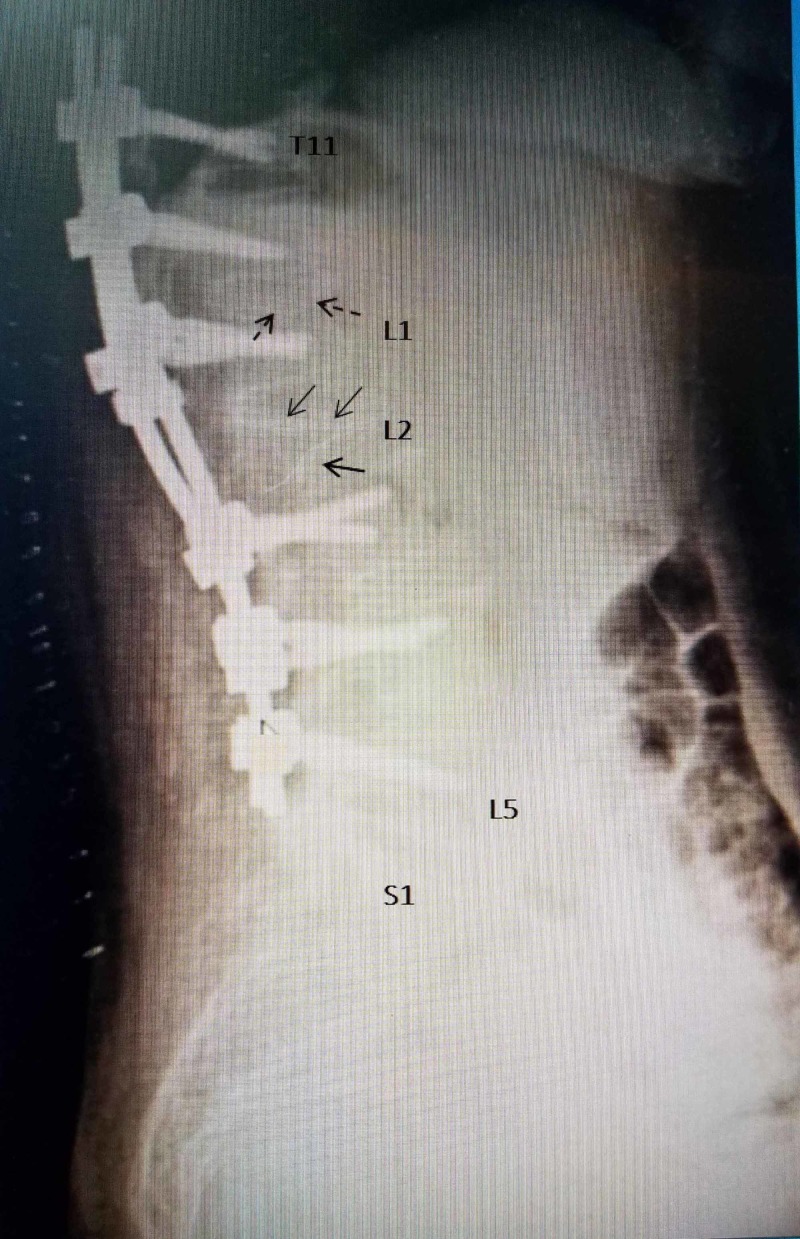
Postoperative lateral X-ray showing screw placement Postoperative lateral radiograph showing the pedicle screws and rods from T11 to L5. The collapsed L2 vertebrae (solid black arrows) is bridged by the screws three segments above and below the fracture and the preoperative kyphosis is reduced closer to normal. The superior endplate of L1 is also partially collapsed (dashed black arrow).

Besides the 70% vertebra plana at L2, there was a superior endplate fracture of L1, so a decision was made to place pedicle screws from T11, T12, and L1, as well as L3, L4, and L5, with long rod fixation providing stabilization over three spinal segments above and below the L2 fracture as well as the area of posterior laminectomy from L1 to L3 required for tumor removal and proper dural repair (Figure 3).

The patient was initially unchanged neurologically in the immediate 48 hours after surgery, but over the next 10 days of the postoperative period, she had full recovery of motor strength, walking without imbalance.

## Discussion

**Figure 4:**
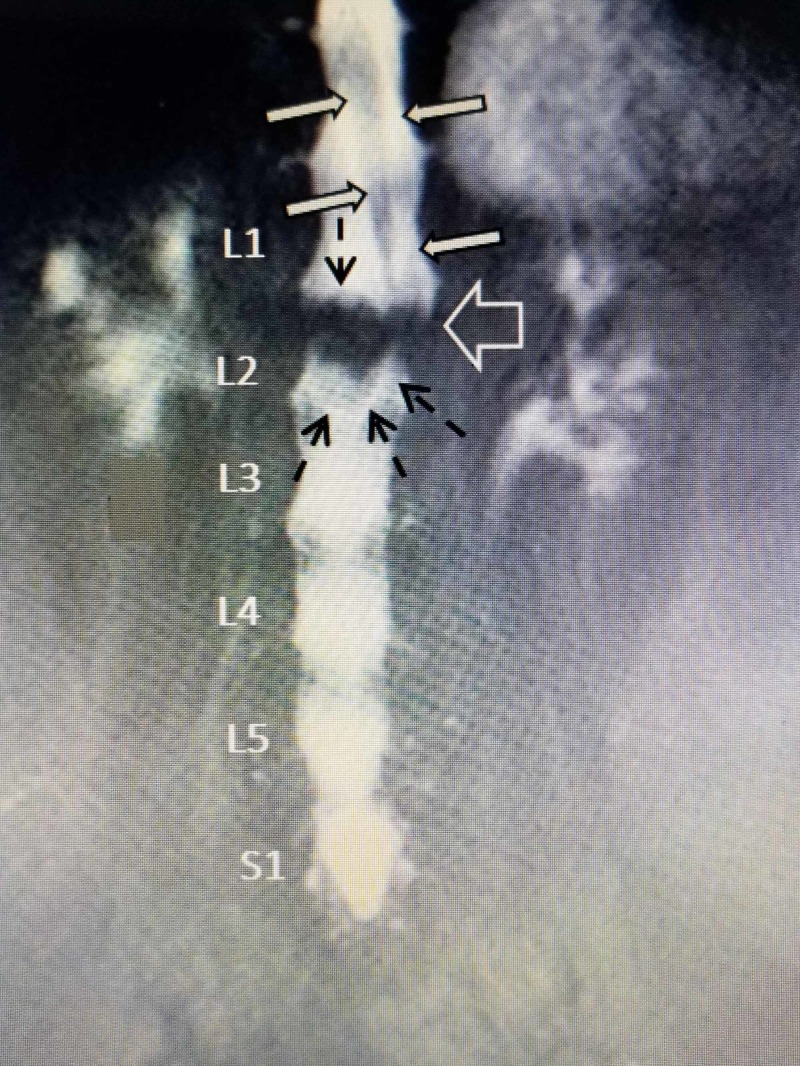
MRI myelogram showing severe localized obstruction of the CSF pattern at the L1-2 level with suggestion of an intraspinal mass The cerebrospinal fluid (CSF) obstruction is clearly seen in this coronal magnetic resonance imaging (MRI) at L1-2 (white border arrow). The upper lumbar nerve roots and conus medullaris are diverted (solid white arrows with black borders) away from a mass (solid black arrows). The outline of an oblong mass on the opposite side of the roots is noted (dashed black arrows).

Although findings of incidental, spinal intradural tumors are well-known, the simultaneous finding of a tumor and a fracture at the same level causing CES is highly unusual [9-10]. CES is a mixture of neurologic symptoms and findings of different levels of severity, including lower extremity weakness, sensory loss, decreased reflexes, and, specifically, incontinence, usually associated with localized spinal stenosis with severe compression of the lumbar nerve roots often seen with larger intraspinal tumors [11-13]. Compression is commonly due to degenerative stenosis or spondylolisthesis, and, after spine trauma, fractures related to either posterior bone displacement or large extruded discs can also cause either neurogenic claudication and eventually CES [14-15]. Studies of both CT and MRI in relationship to CES have determined that it is uncommon to have symptomatic CES with less than 50% obstruction with spinal stenosis [12-13]. Anatomic stenosis with clinical CES is usually very localized as in this case (Figure 4).

The severe spinal canal obstruction in this patient was due to the simultaneous overlapping of two distinct lesions, the intradural, 3-cm oblong tumor, and the acute compression fracture of L2 with dorsal displacement of the superior endplate into the spinal canal at exactly the same level as the tumor. Each could have led to the development of CES separately. In patients with vertebral fractures, displaced endplates and, rarely, large herniated or extruded disc fragments can be associated with CES [15-16]. Severe canal compression with neurological sequelae is more commonly seen with high-velocity burst fractures than low-velocity osteoporotic fractures [16]. Dorsally migrated fragments have also been observed, however, actual neurologic deficits and especially CES is rare without a greater than 50% canal narrowing from the fracture even with kyphotic angulation [13-14]. Rarely, when there is a preexisting narrowed spinal canal from spinal stenosis or even ankylosing spondylitis with a superimposed fracture, CES can develop [15]. Progression of neurologic deficits with the delayed development of CES in burst fractures may indicate fracture expansion or fragment migration [16]. There are also individual case reports of CES with sacral insufficiency fractures affecting the lower sacral roots [17]. In this case, as seen in Figure 1, the sagittal MRI shows that, excluding the intradural tumor, the sagittal narrowing as a result of the posteriorly displaced fractured endplate was less than 33% of the canal and alone would not have likely caused CES.

When spinal tumors grow to a significant size, they present with neurologic complaints and CES [3-5]. Intraspinal tumors make up 15% to 20% of central nervous system tumors, intradural tumors account for 45% of the total, and almost 90% are extra-medullary [3-4]. These tumors are primarily benign, and 85% are either schwannomas, meningiomas, and ependymomas [3,5,9-10]. Benign tumors have a slow rate of growth and clinical symptoms can be very insidious, presenting initially with low back pain and intermittent radiculopathy and are often detected incidentally when an MRI is performed for other reasons [5]. CES is seen in later stages when the tumor has grown enough in size to compromise the spinal canal, leading to the compression of the conus or lower cauda equina roots [3-4,12-13]. Rarely, secondary to torsion of the tumor within the canal, a patient can develop acute symptoms with a previously slow-growing benign tumor [18].

In this patient, wide laminectomy was necessary for dural incision, tumor removal, and dural repair. In this patient with both anterior deformity and instability from the L2 fracture, a small superior endplate fracture at L1 with preoperative kyphosis and then a posterior decompression to remove the tumor, it was essential to perform extensive multi-segmental posterior stabilization [7,11]. This insured there was a minimum of two-segment fixation above and below the ventral fracture (L1 and L2) and dorsal laminectomy. With spinal fractures, recovery of CES is dependent on the length of time of compression, adequate decompression, and fracture reduction with deformity correction [7,19]. As highlighted in this case, with both a large tumor and spinal fracture, recovery of CES is related to preserving the vascular supply to the cauda equina roots as well as complete tumor removal, if possible [3-4,11,16].

## Conclusions

When a patient with an acute spinal fracture presents with clinical findings suggesting the involvement of cauda equina, severe spinal canal compression should be suspected. Studies using both CT and MRI show that in cases with symptomatic CES, spinal canal compression is usually over 50%. In all cases, whether with high-velocity traumatic fractures or, as seen in this case with an osteoporotic fracture after a fall, a careful review of films, examining for any associated pathology, must be done since 'incidental' tumors or other pathology can exist with a fracture. Large disc and endplate fragments, pre-existing degenerative stenosis, spondylolisthesis, ankylosing spondylitis, and, as shown in this rare case, even an unsuspected cauda equina tumor must always be considered in the differential diagnosis when there is a high-grade obstruction. Associated pathology can alter the surgical plan and may necessitate changes in the extent of surgery required.
